# The implications of COP21 for our future climate

**DOI:** 10.1186/s40985-016-0038-z

**Published:** 2016-12-05

**Authors:** Corinne Le Quéré

**Affiliations:** Tyndall Centre for Climate Change Research, University of East Anglia, Norwich, Norfolk NR4 7TJ UK

**Keywords:** Climate change, CO_2_ emissions, Global warming, COP21, Health

## Abstract

Rising CO_2_ in the atmosphere is the main cause of anthropogenic climate change, and the data shows a clear increase in global temperature of about 1 °C since pre-industrial levels. Changes in climate extremes are also occurring, with observed increases in the frequency of heat waves, in intense precipitation (rainfall and snowfall) in many places, and in sea level and storm surges. A changing climate with rising extremes has associated risks for food production and other health-related impacts. In order to limit climate change well below 2 °C, our carbon emissions must rapidly follow a decreasing trajectory to near zero.

## Background

Let us start with the data. The increase in CO_2_ concentration in the atmosphere was first measured directly at Mauna Loa in Hawaii in 1958. The concentration has just crossed 400 ppm (parts per million), an increase of 44 % compared to the pre-industrial levels (around year 1750).

Rising CO_2_ in the atmosphere is the main cause of anthropogenic climate change. To stop the planetary warming, CO_2_ concentration needs to stop rising. In turn to stop the rise in CO_2_ concentration, our carbon emissions must go down to near zero. It is thus no surprise that the global surface temperature has increased, by about 1 °C above pre-industrial levels. However, there are important inter-annual variations in global temperature that are caused by natural climate cycles. For example, the El Niño of 2015–2016 contributed to warming the climate recently, on top of the general trend due to CO_2_ and other greenhouse gases. It is clear though that the climate change trend dominates the recent warming, and our starting point is that human-induced climate change already cause about 1 °C warming. Keep this in mind when we speak about the objectives of the future.

## Main text

Where do we stand as far as CO_2_ emissions are concerned? Emissions must decrease to near zero to stop the rise in atmospheric CO_2_ concentration. We have just published a report which shows that the global emissions of CO_2_ from fossil fuel burning have increased 2–3 % per year on average since the year 2000 [1]. However, the last year of emission data—2014—and our projection for this year—2015—suggest that emission growth has stalled [2]. So a small pause in the long-term emission growth. We are expecting the global emissions to start growing once again, but may be not as fast they have grown since the year 2000. This is good news. The pause in the last 2 years is mainly due to the economic rebalancing in China, with a contribution from the very rapid deployment of renewable energies in the world—a signature of global actions to tackle climate change.

In order to limit climate change well below 2 °C, our emissions must follow a decreasing trajectory to near zero. A large number of scenarios consistent with the two-degree limit include technologies that can actually capture CO_2_ out of the atmosphere and store it below ground. These so-called negative emissions rely on unproven technologies and are in competition with agriculture. They are not a safe bet [3]. At the other extreme, scenarios based on intense use of fossil fuels lead to very high climate change—with a range of related high risks in addition to warming, for example risks of floods from sea level rise and increased heavy rainfall, stress on access to drinking water from salt-water contamination, and droughts, and a range of associated health risks.

What are we expecting from the Paris Agreement on climate change? On the one hand, we have what the countries bring, the ‘NDCs’ for Nationally Determined Contributions. The implementation of the NDCs as they stand would lead to an increase of around 3 °C, somewhere between the 1 °C we are already observing and a planet with a very risky climate future. But the Paris Agreement does set clear ambitions to keep the warming well below 2 °C and to pursue efforts to limit the warming to 1.5 °C, with a roadmap revision for each country every 5 years. There is a conflict between the promised contributions and the level of ambitions, and the outcome for future warming will depend on what individual countries will do next.

We have been working with the World Health Organization (WHO) and other institutes worldwide to do a country-by-country analysis of the implications of climate change [4]. We compared recent temperature observations with warming projections over the country, so people can see the consequences of climate change in their own context. They can relate what a projection of a global temperature rise below 2 °C implies for them compared to a future would in a high-risk climate change. I have spoken a great deal about average temperature, but changes in climate extremes could have the greatest impact on health. Changes in climate extremes have been summarised in a table of the Intergovernmental Panel on Climate Change (IPCC) [5] and the WHO report [4]. Three extremes are particularly clear and well documented: increases in heat waves, increases in intense precipitation (rainfall and snowfall), and increases in sea level and storm surges. The last two have associated increased risks of floods. All have associated risks for food production and possibly pests and disease outbreaks. Even when limiting climate change to 2 °C, understanding regional impacts and adapting to a changing climate will be essential.

## Conclusion

A wise way to respond to the current state of knowledge on climate change would be to prepare to deal with a high-risk climate change future, but to work to mitigate climate change well below 2 °C by reducing global emissions to zero. Adopting this double strategy could help prepare for all eventualities, while working for the outcome with the lowest risks for current and future generations.

## Abbreviations

INDCs: Intended Nationally Determined Contributions
IPCC: Intergovernmental Panel on Climate Change
WHO: World Health Organization
